# Complete Genome Sequence of *Arthrobacter* sp. Strain LS16, Isolated from Agricultural Soils with Potential for Applications in Bioremediation and Bioproducts

**DOI:** 10.1128/genomeA.01586-15

**Published:** 2016-01-14

**Authors:** Ikrema Hassan, Alexander W. Eastman, Brian Weselowski, Eltayeb Mohamedelhassan, Ernest K. Yanful, Ze-Chun Yuan

**Affiliations:** a Southern Crop Protection and Food Research Centre, Agriculture and Agri-Food Canada, London, Ontario, Canada; b Department of Civil and Environmental Engineering, University of Western Ontario, London, Ontario, Canada; c Department of Microbiology and Immunology, University of Western Ontario, London, Ontario, Canada; d Department of Civil Engineering, Lakehead University, Thunder Bay, Ontario, Canada

## Abstract

Here we report the complete genomic sequence of the bacterium *Arthrobacter* sp. strain LS16, consisting of a single circular chromosome of 3.85 Mb with no identified plasmid. Data contained within will facilitate future genetic modification and engineering of the *Arthrobacter* sp. LS16 metabolic network to enhance traits relevant to bioremediation and bioproducts.

## GENOME ANNOUNCEMENT

*Arthrobacter* spp. are soil associated, Gram-positive, obligate aerobes with high survivability that are commonly isolated around the world (1, 2). Various strains have been previously shown to degrade a range of phenolic-derived compounds and are resistant to desiccation, making them desirable for bioremediation (3, 4). *Arthrobacter* sp. strain LS16 (henceforth termed LS16) was isolated from agricultural soils based on its ability to metabolize lignosulfonate, otherwise known as black liquor, a common side product of sulfite pulping. The whole-genome sequence reported here will allow for better understanding of the genetic basis of these traits and bioproductivity.

Genomic DNA of LS16 was isolated from culture grown for 24 h at 37°C in nutrient broth using a Sigma-Aldrich GenElute bacterial genomic DNA kit (product no. NA2120) following the manufacturer’s protocol. A library with an approximate insert size of 500 to 600 bp was prepared for sequencing using a Nextera XT DNA sample preparation kit according to the manufacturers’ protocol. The generated library was used as a template for a limited cycle PCR using Nextera primers, the resulting library was purified, and the quality was evaluated using an Agilent 2100 Bioanalyzer and quantitative PCR (qPCR).

The final library loaded onto the Illumina MiSeq Sequencer at 10 pmol and sequenced using 2- × 300-bp paired-end chemistry generating 7.62 million read pairs representing 100× coverage of the LS16 genome. Read pairs were trimmed, resulting in a 94× coverage library, and the reads were assembled *de novo* and scaffolded using SPAdes (K-mer values of 21, 33, 55, 77, 99, and 127) resulting in a draft assembly of 33 contigs (5). This assembly had a largest contig size of 798 kb, *N*_50_ length of 347 kb, and total genome size of 3.85 Mb. Finishing of the contig gaps was done using long and accurate PCR across gaps with primers complementary to protein coding sequences at the 5′ and 3′ ends of contigs, followed by Sanger sequencing and primer walking where necessary as described previously (6).

The genome of LS16 consists of a single circular chromosome of 3,851,242 bp, with a G+C content of 64.32%. Annotation was completed using the NCBI Prokaryote Genome Annotation Pipeline (7–9). Annotation revealed 3,551 genes, 3,255 coding sequences (CDS), 209 pseudogenes, 1 ncRNA, 31 frameshifted genes, 67 tRNA loci, and 19 rRNAs genes, which is in accordance with other completely sequenced *Arthrobacter* sp. genomes. The closest related relative of LS16 is *Arthrobacter arilaitensis* RE117, with variable levels of synteny between the strains (10).

LS16 demonstrates various characteristics amicable to industrial applications, including metabolism of lignin-derived and phenolic compounds, robust growth at low pH, and bioremediation of contaminated soils. In-depth analysis of the *Arthrobacter* sp. LS16 genome will further our understanding of the underlying mechanisms guiding genetic modification of pathways to enhance bioremediation traits.

### Nucleotide sequence accession number.

The whole-genome sequencing project of *Arthrobacter* sp. LS16 has been deposited in GenBank under the accession number CP012171. The version described in this paper is the first version.

## References

[1] Mongodin EF, Shapir N, Daugherty SC, DeBoy RT, Emerson JB, Shvartzbeyn A, Radune D, Vamathevan J, Riggs F, Grinberg V, Khouri H, Wackett LP, Nelson KE, Sadowsky MJ 2006 Secrets of soil survival revealed by the genome sequence of *Arthrobacter aurescens* TC1. PLoS Genet 2:e214. doi:10.1371/journal.pgen.0020214. 17194220 PMC1713258

[2] Hagedorn C, Holt JG 1975 A nutritional and taxonomic survey of *Arthrobacter* soil isolates. Can J Microbiol 21:353–361. doi:10.1139/m75-050. 1116046

[3] Unell M, Nordin K, Jernberg C, Stenström J, Jansson JK 2008 Degradation of mixtures of phenolic compounds by *Arthrobacter chlorophenolicus* A6. Biodegradation 19:495–505. doi:10.1007/s10532-007-9154-2. 17917705

[4] Pieper DH, Reineke W 2000 Engineering bacteria for bioremediation. Curr Opin Biotechnol 11:262–270. doi:10.1016/S0958-1669(00)00094-X. 10851148

[5] Bankevich A, Nurk S, Antipov D, Gurevich AA, Dvorkin M, Kulikov AS, Lesin VM, Nikolenko SI, Pham S, Prjibelski AD, Pyshkin AV, Sirotkin AV, Vyahhi N, Tesler G, Alekseyev MA, Pevzner PA 2012 SPAdes: A new genome assembly algorithm and its applications to single-cell sequencing. J Comput Biol 19:455–477. doi:10.1089/cmb.2012.0021. 22506599 PMC3342519

[6] Eastman AW, Yuan Z 2015 Development and validation of an rDNA operon based primer walking strategy applicable to *de novo* bacterial genome finishing. Front Microbiol 5:769. doi:10.3389/fmicb.2014.00769. 25653642 PMC4301005

[7] Angiuoli SV, Gussman A, Klimke W, Cochrane G, Field D, Garrity GM, Kodira CD, Kyrpides N, Madupu R, Markowitz V, Tatusova T, Thomson N, White O 2008 Toward an online repository of standard operating procedures (SOPs) for (meta)genomic annotation. OMICS 12:137–141. doi:10.1089/omi.2008.0017. 18416670 PMC3196215

[8] Besemer J, Lomsadze A, Borodovsky M 2001 GeneMarkS: a self-training method for prediction of gene starts in microbial genomes. Implications for finding sequence motifs in regulatory regions. Nucleic Acids Res 29:2607–2618. doi:10.1093/nar/29.12.2607. 11410670 PMC55746

[9] Gao F, Zhang C 2008 Ori-finder: a web-based system for finding oriCs in unannotated bacterial genomes. BMC Bioinformatics 9:79. doi:10.1186/1471-2105-9-79. 18237442 PMC2275245

[10] Monnet C, Loux V, Gibrat J, Spinnler E, Barbe V, Vacherie B, Gavory F, Gourbeyre E, Siguier P, Chandler M, Elleuch R, Irlinger F, Vallaeys T 2010 The *Arthrobacter arilaitensis* Re117 genome sequence reveals its genetic adaptation to the surface of cheese. PLoS One 5:e15489. doi:10.1371/journal.pone.0015489. 21124797 PMC2991359

